# PReS-FINAL-2243: Reduced volumetric trabecular bone mineral density in children with idiopathic hypercalciuria. A peripheral quantitative computed tomography (PQCT) study (preliminary results)

**DOI:** 10.1186/1546-0096-11-S2-P233

**Published:** 2013-12-05

**Authors:** E Atsali, KD Stathopoulos, I Bournazos, P Nicolaidou, P Papaggelopoulos, G Skarantavos

**Affiliations:** 1 "Attikon" University Hospital, Haidari - Athens, Greece

## Introduction

Idiopathic hypercalciuria (IH) is defined as excessive 24 h urinary calcium excretion (>4 mg/kg/24h), that persists after correction of dietary imbalances, in the absence of secondary causes. Decreased areal BMD in children with IH has been reported in recent studies with DXA.

## Objectives

We used peripheral Quantitative Computed Tomography (pQCT) of the tibia in order to test the hypothesis that IH results in decreased volumetric (mg/cm^3^) BMD of trabecular and/or cortical compartment of bone.

## Methods

We studied 14 children (8 boys- 6 girls, aged 6-18 years) with newly discovered IH who were admitted to our clinic. Most of them presented with either hematuria or recurrent abdominal or lumbar pain. After establishment of the diagnosis, all children underwent DXA of the lumbar spine. We also performed pQCT of the tibia (Stratec XCT-2000 scanner), 4 slices were obtained at the 4%, 14%, 38% and 66% of tibia length sites. For the 4% slice, we assessed variables of trabecular bone and especially trabecular BMD (TRAB_DEN, mg/cm^3^). For 14% we assessed parameters of subcortical bone and for the 38% and 66% sites parameters of cortical bone. pQCT data of the children with IH were compared to those of healthy race-, age- and sex-matched children from the published pQCT database of Moyer-Miller et al (J Clin Densitom, 2008) who used the same pQCT device, software and site measurements as we did.

## Results

7/14 children with IH (50%) were found to have z-scores<-1 SD in the DXA measurements of the lumbar spine. For the pQCT measurements, we report here only the preliminary results of trabecular BMD (ongoing analysis): 8/14 children with IH (57%) had reduced volumetric bone mineral density (TRAB_DEN < 2 SD) when compared with healthy children of the same age, race, sex and height of the Moyer-Miller study.

## Conclusion

Our study provides preliminary evidence of reduced trabecular bone mineral density in children with IH as compared to healthy ones.

## Disclosure of interest

None declared.

